# Rapid and Low-Cost Quantification of Adulteration Content in *Camellia* Oil Utilizing UV-Vis-NIR Spectroscopy Combined with Feature Selection Methods

**DOI:** 10.3390/molecules28165943

**Published:** 2023-08-08

**Authors:** Qiang Liu, Zhongliang Gong, Dapeng Li, Tao Wen, Jinwei Guan, Wenfeng Zheng

**Affiliations:** School of Mechanical and Electrical Engineering, Central South University of Forestry and Technology, Changsha 410004, China; qiangliu999928@163.com (Q.L.); gzlaa@163.com (Z.G.); twen@csuft.edu.cn (T.W.); guanjinwei330124@163.com (J.G.); thenwinfor@163.com (W.Z.)

**Keywords:** *Camellia* oil, UV-Vis-NIR spectroscopy, adulteration content prediction, chemometrics, feature wavelength selection, absorbance, correlation analysis

## Abstract

This study aims to explore the potential use of low-cost ultraviolet-visible-near infrared (UV-Vis-NIR) spectroscopy to quantify adulteration content of soybean, rapeseed, corn and peanut oils in *Camellia* oil. To attain this aim, test oil samples were firstly prepared with different adulterant ratios ranging from 1% to 90% at varying intervals, and their spectra were collected by an in-house built experimental platform. Next, the spectra were preprocessed using Savitzky–Golay (SG)–Continuous Wavelet Transform (CWT) and the feature wavelengths were extracted using four different algorithms. Finally, Support Vector Regression (SVR) and Random Forest (RF) models were developed to rapidly predict adulteration content. The results indicated that SG–CWT with decomposition scale of 2^5^ and the Iterative Variable Subset Optimization (IVSO) algorithm can effectively improve the accuracy of the models. Furthermore, the SVR model performed best for predicting adulteration of camellia oil with soybean oil, while the RF models were optimal for camellia oil adulterated with rapeseed, corn, or peanut oil. Additionally, we verified the models’ robustness by examining the correlation between the absorbance and adulteration content at certain feature wavelengths screened by IVSO. This study demonstrates the feasibility of using low-cost UV-Vis-NIR spectroscopy for the authentication of *Camellia* oil.

## 1. Introduction

*Camellia* oil, as one of the most valuable edible oils [1,2], is highly sought after by consumers because of its great potential in the health care, medical, beauty, and chemical fields [3,4]. The superior physicochemical properties possessed by *Camellia* oil result in its price being significantly higher (5–10 times) than that of common edible oils (such as soybean and rapeseed oils) [5]. Due to the high demand, several fraudulent practices have emerged in the *Camellia* oil industry. These involve illegal traders who blend low-priced oils, such as soybean, corn, and rapeseed oils, into the genuine *Camellia* oil, as well as falsely label their product to make illicit profits [6]. Such practices are serious violations of regulations such as Chinese standard GB 2716-2018. It is therefore critical to develop a rapid, effective and robust method for quantifying adulteration content in *Camellia* oil.

Traditional methods for detecting adulteration in *Camellia* oil based on visual or odor assessments are often subjective, leading to unreliable results [7]. Recently, primary methods for detecting edible oil adulteration have included chromatography [8], nuclear magnetic resonance [9], electronic nose [10], and spectroscopy [11]. Among them, chromatography, nuclear magnetic resonance, and the electronic nose method have certain drawbacks. For example, gas chromatography and liquid chromatography require complex chemical pretreatment, thus leading to extended detection periods [8,11]. Moreover, nuclear magnetic resonance needs expensive equipment and complex operation [9,12], while electronic nose often faces the issue of unstable data acquisition [10]. Various spectroscopy techniques have been applied to authenticate adulterated *Camellia* oil thanks to their direct and non-destructive characteristics [13,14,15]. However, most studies in this field typically involve the use of expensive near-infrared (NIR) spectrometers to acquire the data [5,11,16]. Moreover, the majority of studies have been performed by either using the entire spectrum bands or solely relying on the feature wavelength bands [17,18]. Therefore, these have led to issues such as high detection costs and a lack of ability to merge the rapidity and robustness of model authentication.

Studies that examine the authentication of edible oil using UV-Vis spectroscopy, which has significant cost advantages compared with NIR spectroscopy, have recently been increasing. Su et al. [19] utilized an in-house built acquisition platform to collect reflectance spectra of 110 samples of sesame and rapeseed oils adulterated with soybean oil, and developed the optimal models to predict their adulteration content. However, the developed models were not effective for low percentage adulteration content (i.e., less than 10%) [19]. Wu et al. [20] utilized UV-Vis spectroscopy (350–800 nm) to create binary and ternary quantitative models with weighted multiscale support vector regression (EMD-SVR) for soybean, peanut, and sesame oils. This study demonstrates that UV-Vis spectroscopy could be employed for efficient authentication of edible oils. Gong et al. [21] investigated the qualitative detection of adulterated *Camellia* oil by utilizing UV-Vis-NIR transmission spectroscopy (200–1100 nm). They established an XGBoost model, which can identify *Camellia* oil adulterated with sunflower, corn and peanut oils, respectively, with high accuracy (98.18%), sensitivity (97.62%), and specificity (100%). Although the abovementioned study elucidated that UV-Vis-NIR spectroscopy can be utilized for the authentication of edible oils, quantitative detection of adulteration content in *Camellia* oil through this technique has not yet been reported.

To fill this gap, this paper presented a study on quantitative evaluation of adulteration content in *Camellia* oil using UV-Vis-NIR transmission spectroscopy (200–1100 nm). The study involved the comparison of various spectrum preprocessing, feature wavelength selection, and machine learning modeling methods to establish rapid and robust models for quantifying the adulteration content of soybean, rapeseed, corn, and peanut oils, respectively, in *Camellia* oil. In addition, the rationality of the obtained feature wavelengths was verified through Poisson correlation analysis, further proving the reliability of the established models. Consequently, this study lays a foundation to develop a low-cost, rapid and robust quantification device for the authentication of *Camellia* oil.

## 2. Results and Discussion

### 2.1. Spectrum of the Oil Samples

**Figure 1:**
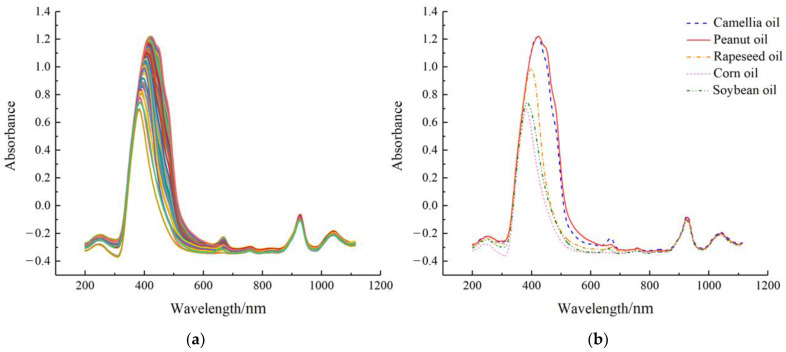
Raw spectra of the samples: (**a**) all samples; (**b**) representative pure oil samples.

The raw spectrum profiles of the prepared samples were presented in Figure 1. As shown in Figure 1a, there were five absorption peaks in the 244 spectrum curves at around 250 nm (UV band), 430 and 660 nm (Vis band), and 930 and 1050 nm (NIR band), respectively. Figure 1b illustrated that there were differences between the spectrum curves of the five pure oils, particularly in some bands where *Camellia* oil differed from the four lower-priced oils. Specifically, at the characteristic peak band near 430 nm, the largest magnitude differences between *Camellia* oil and rapeseed, corn, and soybean oils were observed. Additionally, a significant magnitude difference existed between *Camellia* oil and peanut oil at the characteristic peak band around 660 nm. The observed differences between the collected pure oil spectrum provided evidence of utilizing UV-Vis-NIR transmission spectroscopy (200–1100 nm) as a feasible method to estimate adulterated content. However, due to the similarity between spectra curves, it is necessary to employ chemometrics and machine learning techniques to further analyze the data.

### 2.2. Spectrum Preprocessing Results

**Figure 2:**
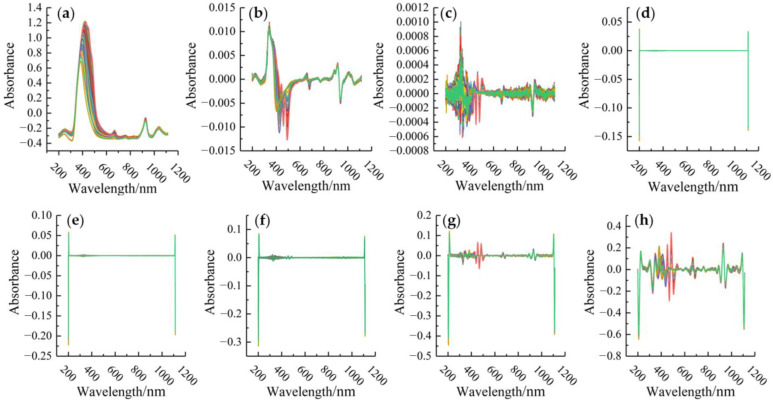

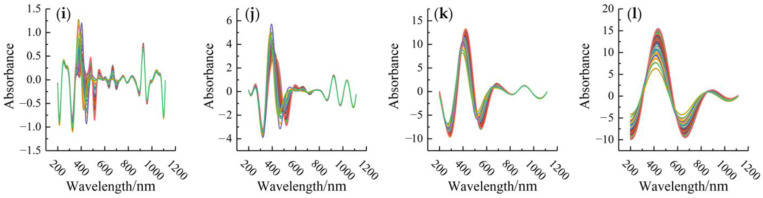
Spectra pre-processing results. (**a**) SG; (**b**) SG-1st; (**c**) SG-2nd; (**d**–**l**) multi-decomposition scale SG–CWT from L1 to L9, respectively.

The raw spectra were preprocessed with SG, SG-1st, SG-2nd, and SG-CWT with 9 decomposition scales (i.e., L1–L9), respectively (Figure 2).

To determine the optimal preprocessing method, the SVR models were developed using the full preprocessed spectral dataset, and then R^2^ and RMSE were evaluated. As shown in Table 1, the optimal method was SG–CWT (L5) with an $R^{2}$ of 0.9998 and RMSE of 0.0059. Moreover, compared to SG-2nd that was the second-best method, SG–CWT (L5) can improve RMSE by 21.33% and R^2^ by 0.02%. In addition, the results verified that SG–CWT (L1−L9) was able to gradually amplify the insignificant characteristic peaks and valleys in the spectral curves at low decomposition scales, thus highlighting the local differences (Figure 2). However, increasing decomposition scale led to too smooth spectral curves, and hence, some inconspicuous feature peaks and valleys were gradually removed, which increased the difficulty of capturing spectral feature information. It was demonstrated that CWT can effectively smooth the data and reduce noise at low decomposition scales. Therefore, SG–CWT (L5) was adopted to preprocess spectra for the following parts.

### 2.3. Feature Wavelength Screening Results

Four kinds of wavelength screening methods, including CARS, SPA, BOSS, and IVSO, were used to select the feature wavelengths from the full spectrum with SG–CWT (L5) preprocessing. As a result, CARS, SPA, BOSS, and IVSO screened 27, 28, 30, and 48 feature wavelengths, respectively, effectively reducing the number of wavelengths to 1.31%, 1.35%, 1.45%, and 2.32% of the full spectrum (2068 wavelengths).

**Figure 3:**
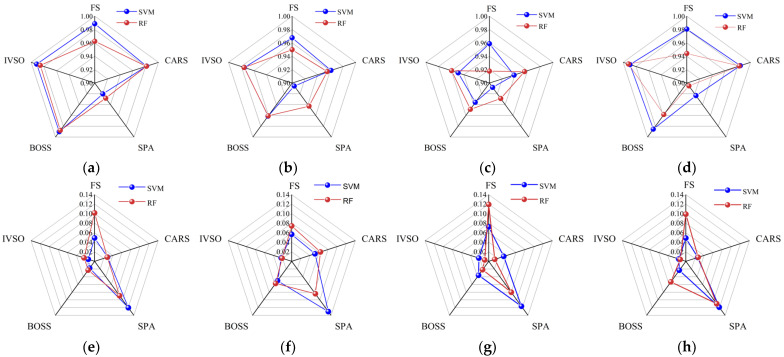
Comparison of evaluation criteria for different feature wavelength screening methods. (**a**–**d**): the $R_{P}^{2}$ of *Camellia* oil adulterated with soybean, corn, peanut, and rapeseed oils, respectively; (**e**–**h**): the ${\mathrm{RMSE}}_{P}$ of *Camellia* oil adulterated with soybean, corn, peanut, and rapeseed oils, respectively.

To determine the optimal feature wavelength screening method, the SVR and RF-based models were established using full spectrum (FS) and the feature wavelengths screened by CARS, SPA, BOSS and IVSO, respectively. Then, the evaluation criteria of different models were compared in Figure 3. The results demonstrated that the IVSO method was the most appropriate for feature wavelength screening, trailed by BOSS and CARS, while SPA was the least suitable. Furthermore, the results indicated that the use of IVSO-RF provided the optimal performance for quantifying the percentage of adulterant in *Camellia* oil adulterated with corn, peanut, or rapeseed oils. However, for *Camellia* oil adulterated with soybean oil, the best performance was achieved using the IVSO-SVR combination.

**Figure 4:**
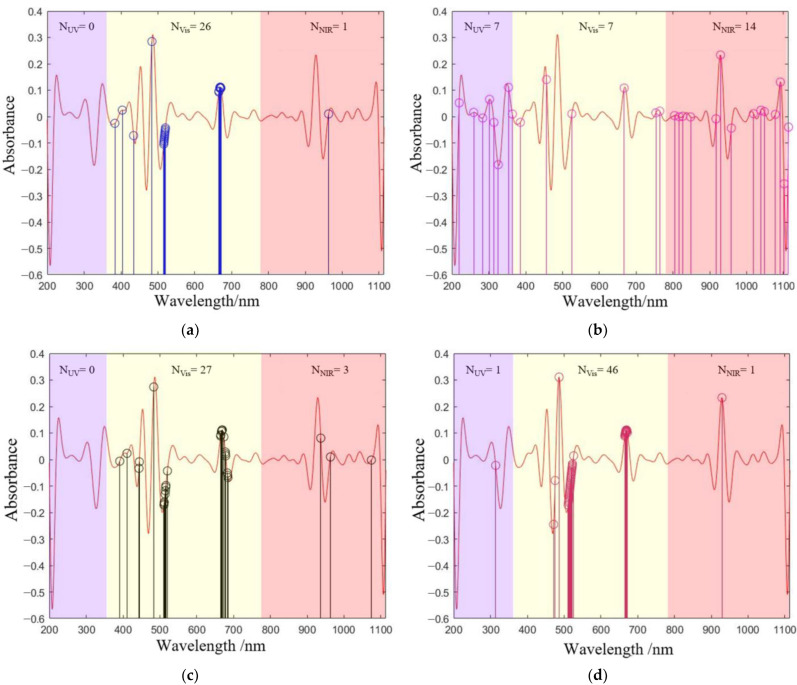
Distribution of feature wavelengths screened by (**a**) CARS, (**b**) SPA, (**c**) BOSS and (**d**) IVSO.

To further clarify the advantages of IVSO compared to other methods, the distribution characteristics of the screened wavelengths were analyzed (Figure 4). The wavelengths extracted by CARS, BOSS, and IVSO were predominantly in the Vis band, while SPA had comparable numbers of wavelengths among the UV, Vis and NIR bands. Specifically, CARS, BOSS, and IVSO concentrated the feature wavelengths on two characteristic peak and valley positions at approximately 510 nm and 660 nm. In contrast, the feature wavelengths generated by SPA were primarily concentrated at both ends of the spectrum, where excessive noise interference could render SPA a distinct disadvantage over the other methods. In addition, IVSO showed better clustering characteristics than CARS and BOSS in the Vis band.

### 2.4. Model Development

In this study, we developed an SVR model to predict the adulteration content of soybean oil mixed with *Camellia* oil, and three RF models to measure adulteration with corn, peanut, and rapeseed oils, respectively, according to the results shown in Figure 3.

Moreover, to further enhance the prediction ability of the proposed models, the main parameters in SVR and RF models, as presented in Table 2, were optimized. Specifically, the SVR model determined the optimal penalty factor *c* and the kernel function *γ* by the grid search method that minimized 10-fold cross-validation error. Moreover, the RF models attained the optimal number of decision trees *n* by using the particle swarm optimization algorithm, which was run with a particle dimension of 2, population size of 30, and maximum 100 iterations. The performance of both SVR and RF models were also shown in Table 2.

As presented in Table 2, it was apparent that the parameter-optimized models significantly outperformed their unoptimized counterparts (Figure 3). Additionally, the SVR and RF models demonstrated no signs of underfitting or overfitting.

Next, a detailed analysis of each of the four models was presented. The analysis utilized the evaluation criteria for prediction sets and provided insight into each model’s performance. The $R_{P\;}^{2}$ values indicated that the parameter-optimized models performed better in each of the four types of adulterated oils. Specifically, in the case of *Camellia* oil adulterated with soybean oil, the parameter-optimized IVSO-SVR model achieved a higher $R_{P\;}^{2}$ of 0.9925 (an improvement of 0.13%), and a lower ${\mathrm{RMSE}}_{P}$ of 0.0325 (a decrease of 8.96%) compared to the unoptimized IVSO-SVR model (0.9912 and 0.0357, respectively). The same trend held true for the parameter-optimized IVSO-RF model in the other three types of adulterated oils, exhibiting an increase in $R_{P\;}^{2}$ of 0.92% to 0.19% and a decrease in ${\mathrm{RMSE}}_{P}$ of 10.14% to 18.94% when compared to unoptimized models.

**Figure 5:**
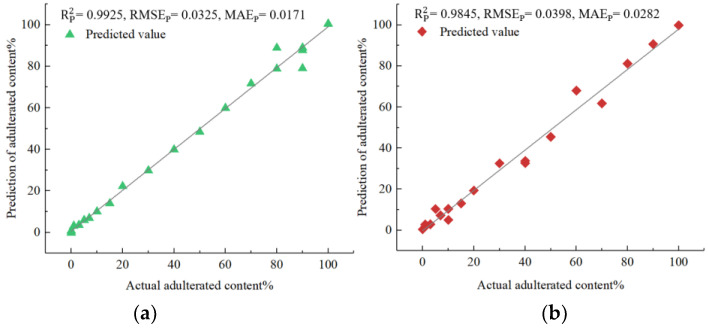

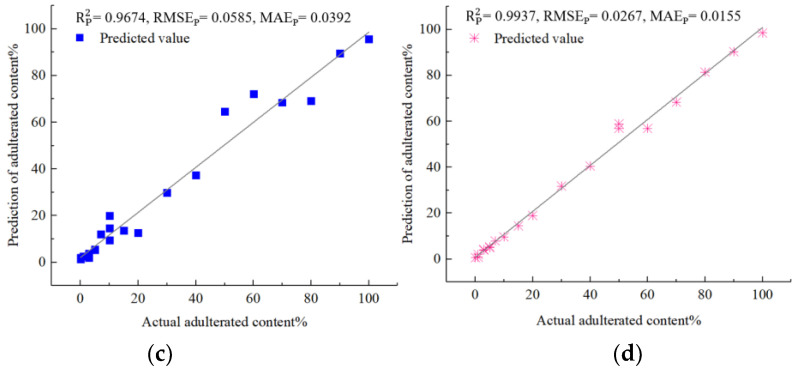
Comparison of predicted and actual adulteration content for (**a**) *Camellia* oil adulterated with soybean oil; (**b**) *Camellia* oil adulterated with corn oil; (**c**) *Camellia* oil adulterated with peanut oil; (**d**) *Camellia* oil adulterated with rapeseed oil.

Figure 5 illustrates that the predicted adulteration content of soybean, corn, peanut and rapeseed oils all agreed well with actual ones in terms of prediction sets. Therefore, these results strongly indicated that the developed models can greatly assist in quantifying the adulteration content in *Camellia* oil with high precision and reliability. Furthermore, the performance of the developed models was compared with that of other spectroscopic quantitative techniques including Vis-NIR [19], Fourier transform infrared (FTIR) [22] and UV-Vis [15] (Table 3). It was observed that our models demonstrated comparable predictive capabilities, while providing a broader range for adulteration ratios.

### 2.5. Wavelength Characteristics Study

In order to further emphasize the applicability of the models established in this study and the rationality of the selected feature wavelengths, three representative wavelengths among the feature wavelengths proposed by IVSO were analyzed by significance test and Pearson correlation coefficient. They were 313.759 nm in the UV band, 486.401 nm in the Vis band, and 929.144 nm in the NIR band. The significance coefficients ρ for these three wavelengths were all less than 0.05, and therefore, possessed the significance of Pearson correlation analysis for the study [23].

**Figure 6:**
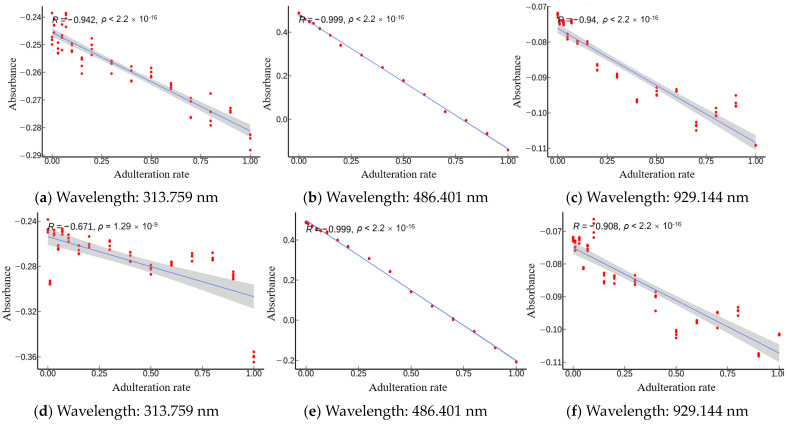

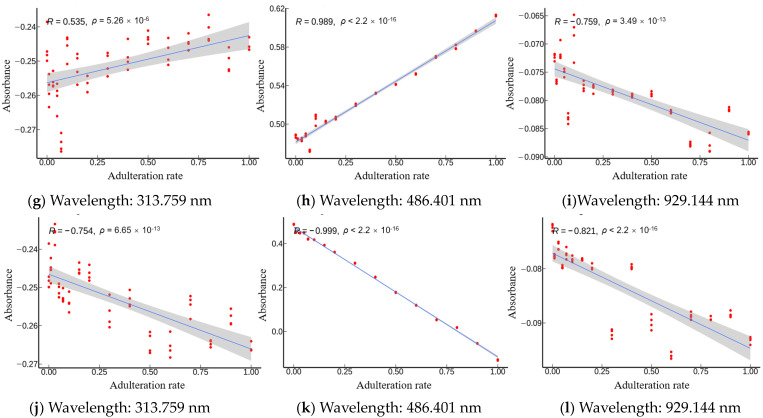
Pearson correlation analysis between the absorbance (at the wavelengths of 313.759 nm, 486.401 nm and 929.144 nm) and adulteration content of (**a**–**c**) soybean oil; (**d**–**f**) corn oil; (**g**–**i**) peanut oil; (**j**–**l**) rapeseed oil.

We compared the Pearson correlation coefficients across three bands for *Camellia* oil adulterated with four oils (Figure 6). It was found that the absolute values of coefficients |R| were all greater than 0.5. This suggested there were some correlations between the absorbance and adulteration content. Furthermore, Figure 6 displays the Pearson correlation coefficient of absorbance and adulteration content in decreasing order of strength from Vis, NIR, and UV. This supports the possibility that the inadequate number of Vis wavelengths may have an adverse effect on the predictive capability of the SPA algorithm.

Specifically, *Camellia* oil that is adulterated with soybean oil (Figure 6a–c) exhibited negative correlation across all three wavelengths with an R value less than −0.94. This implied that accurate prediction of the adulteration content of soybean oil can be made in the wavelength bands of 200–1100 nm. Next, the *Camellia* oil adulterated with corn oil (Figure 6d–f) displayed negative correlation in all three bands. The Vis and NIR bands showed a high level of correlation (with R value less than −0.90), whereas the UV band demonstrated a moderate correlation with an R value of −0.671. This indicated that the Vis and NIR bands contained more feature information than the UV band. In addition, the adulteration of *Camellia* oil with peanut oil (Figure 6g–i) displayed positive correlation for the UV and Vis wavelengths, but showed negative correlation in the NIR band. R values of 0.989 (very strong correlation), −0.759 (strong correlation), and 0.535 (moderate correlation) were obtained for the Vis, NIR, and UV bands, respectively. This indicated that the Vis wavelength contained more sensitive information in the adulteration of peanut oil. Last, in the instance of *Camellia* oil that is adulterated with rapeseed oil (Figure 6j–l), negative correlation was observed across all three bands, with Vis and NIR bands exhibiting very strong correlation (R values less than −0.82), while a strong correlation (R value of 0.754) was exhibited by the UV band. This demonstrated that the prediction ability of the Vis and NIR bands was stronger than that of the UV band in the case of rapeseed oil adulteration. In summary, the IVSO-based feature extraction method was demonstrated to be robust.

## 3. Materials and Methods

The overall research process diagram is shown in Figure S1 of Supplementary Material.

### 3.1. Samples Preparation

Five different types of oils were purchased from the local supermarkets in Changsha, China, including *Camellia*, soybean, rapeseed, corn, and peanut oils. All oils used during the test were within the shelf life.

Soybean, rapeseed, corn and peanut oils were blended into *Camellia* oil at different blend ratios to prepare adulterated samples. The proportion of blend was 1%, 3%, 5%, 7%, 10%, 15%, 20%, 30%, 40%, 50%, 60%, 70%, 80%, 90% respectively. Four samples were prepared for both each blend ratio of the adulterated samples and five kinds of pure oils. Consequently, the number of test samples in each set of adulterated oil samples reached 64, while the total number of samples in this study was 256. For preparation, the mixed oil samples were placed in a magnetic mixer and stirred at 35 °C for 1 h, followed by a low temperature resting period of 24 h.

### 3.2. Experimental Platform

**Figure 7:**
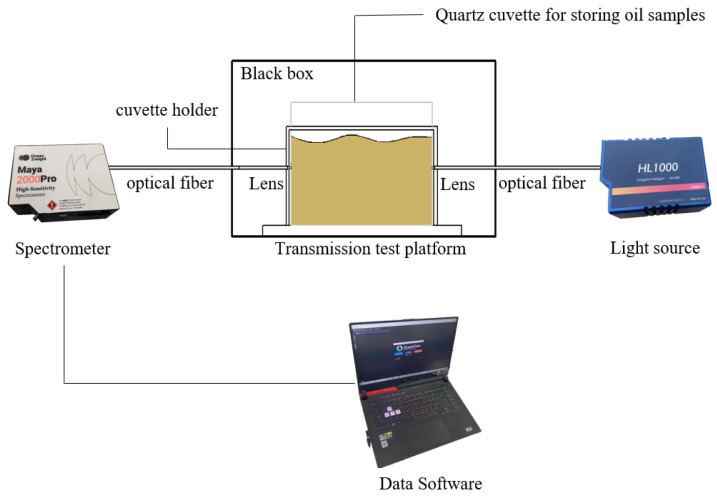
Transmission measurement platform.

An experimental platform (Figure 7) for collecting transmission spectra was composed of a dark box, a cuvette holder, two probe holders, two optical fibers, a spectrometer (OceanView Maya2000 pro, OceanView Company of the United States, Phoenix, AZ, USA), and a light source (HL1000 tungsten halogen lamp, Wen Yi Optoelectronics Technology Co., Shanghai, China). The spectra were collected by OceanView spectroscopy software.

The spectrometer has a spectrum scan range of 200–1100 nm, and was configured to the spectrum integration time of 32 ms and a number of scans of 100. Prior to the experiment, the spectrometer was preheated for 40 min. The mean value was obtained by measuring the same sample three times and calculating the average. The absorbance of the samples was calculated by Equation (1).

(1) $$\begin{array}{c} A_{\lambda }=-Log_{10}\left(\frac{S_{\lambda }-D_{\lambda }}{R_{\lambda }-D_{\lambda }}\right) \end{array}\;\;\;\;$$

where $A_{\lambda }$ is the absorbance, $S_{\lambda }$ is the intensity of the collected sample spectrum, $D_{\lambda }$ is the background intensity of the dark spectrum, and $R_{\lambda }$ is the intensity of the empty cuvette reference spectrum.

### 3.3. Spectrum Preprocessing

The spectrum acquisition process is highly susceptible to interference from the external environment or its own internal instability, thus causing confusion and loss of valid information in the spectrum data. To improve the model accuracy, Savitzky–Golay (SG), SG-1st, SG-2nd, and SG-multi-decomposition scale Continuous Wavelet Transform (CWT) were used to preprocess the raw spectra in this study. SG smoothing can effectively improve the signal-to-noise ratio and also has a significant effect on reducing random noise. In this study, the window size for the SG was set to 15 and the polynomial degree was set to 2. Setting the window size too large would lead to excessive smoothing of the spectrum curve, while setting it too small would result in ineffective noise removal [24]. First-order and second-order derivative processing are effective methods for resolving overlapping peaks and eliminating background noise [24]. CWT has the property of refining weak information to highlight localization and can effectively enhance spectrum feature information, and has been applied in noise reduction, de-contextualization, and compression of spectrum data [25,26]. The decomposition scales in CWT were set to 2, $2^{2}$, $2^{3}$, $2^{4}$, $2^{5}$, $2^{6}$, $2^{7}$, $2^{8}$, and $2^{9}$, which were chosen as the mother wavelet functions for the identification of the dopant content spectra. For simplification, the nine decomposition scales were designated as L1–L9, respectively [25,26].

The preprocessed spectra were brought into the SVR prediction model, and then the optimal preprocessing method was selected by comparing the evaluation criteria of coefficient of determination (R^2^) and root mean square error (RMSE).

### 3.4. Feature Wavelength Selection Method

The full spectrum has 2068 wavelengths, and the number of redundant wavelengths affects the accuracy and speed of model predictions. To simplify the prediction model, the feature wavelengths of the preprocessed data were selected by Competitive Adaptive Reweighted Sampling (CARS), Successive Projections Algorithm (SPA), Bootstrapping Soft Shrinkage (BOSS), and Iteratively Variable Subset Optimization (IVSO), respectively.

CARS screens the subset of wavelengths with the smallest root mean square error of cross-validation (RMSECV) as the feature wavelength set with a 10-fold interaction validation cycle [27]. The number of Monte Carlo runs was set to 100 in this study, and 80% of the samples were taken each time as the calibration set.

SPA minimizes the spectrum data dimensionality by reducing the number of input features [28]. The range of the number of wavelengths after setting the SPA downscaling in this study was from 1 to 40.

BOSS continuously adjusts the weights of each wavelength by weighted autonomous sampling (WBS), which can achieve optimal shrinkage of wavelength space [29]. The number of bootstrap samples was set to 1000 in this study.

IVSO simulates the competitive process by weighted binary matrix sampling (WBMS) and sequential addition to progressively reduce the number of useless and interfering information wavelengths [30]. The number of WBMS samples was set to 1000 in this study.

### 3.5. Models

Support Vector Regression (SVR) and Random Forest (RF) algorithms were utilized to develop the rapid quantitative model of adulterated *Camellia* oil based on extracted feature wavelengths. Before constructing models, the Kennard–Stone (K–S) algorithm [31] was used to divide the datasets into two segments in a 2:1 ratio, resulting in 43 samples in calibration set and 21 samples in prediction set for each type of adulterated oil samples. Furthermore, the model’s performance was improved through the use of a parameter search method.

SVR constructs the optimal hyperplane to solve the optimal solution by selecting different kernel functions so that the features are mapped to the high-dimensional space. The radial basis function in the kernel function is a nonlinear projection that can cope better with the nonlinear relationship between the label and the predictor variables [32]. In this study, the radial basis function was chosen as the kernel function to develop the SVR model for the fast prediction of adulteration content within *Camellia* oil. The penalty factor *c* and kernel function *γ* within the SVR model were optimized using the grid search method [33] to improve its ability to predict adulteration content within *Camellia* oil.

RF is an ensemble learning method that utilizes decision trees to make a collective decision, where the majority vote determines the final results [34]. The number of decision trees is a crucial parameter that influences the accuracy of RF. To improve the performance of RF model in predicting the adulterated content of *Camellia* oil, a particle swarm optimization algorithm [35] was utilized to determine the optimal number of decision trees.

### 3.6. Model Evaluation

This study evaluated the performance of the developed models by using the coefficient of determination (R^2^), mean absolute error (MAE), and root mean square error (RMSE) [36], which were defined as Equations (2)–(4), respectively. The accuracy of the models was measured by R^2^, which ranges from 0 to 1. A lower value of RMSE indicates a better agreement between the predicted and actual adulteration content. Moreover, MAE is a better measure than RMSE for evaluating model accuracy in the presence of outliers. In addition, the calibration set results in the evaluation criteria are expressed as $R_{C}^{2}$, ${\mathrm{RMSE}}_{C}$, ${\mathrm{MAE}}_{C}$, respectively, while the prediction set results are expressed as $R_{P}^{2}$, ${\mathrm{RMSE}}_{P}$, ${\mathrm{MAE}}_{P}$, respectively.

(2) $$R^{2}=1-\frac{\sum_{i=1}^{n}{\left(y_{i}-{\hat{y}}_{i}\right)}^{2}}{\sum_{i=1}^{n}{\left(y_{i}-\bar{y}\right)}^{2}}$$

(3) $$\mathrm{RMSE}=\sqrt{\frac{1}{n}\overset{n}{\underset{i=1}{\sum }}{\left(y_{i}-{\hat{y}}_{i}\right)}^{2}}$$

(4) $$\mathrm{MAE}=\frac{1}{n}\overset{n}{\underset{i=1}{\sum }}|y_{i}-{\hat{y}}_{i}|$$

where, y and y^ denote real and predicted adulteration content, respectively; $\overset{¯}{y}$ denotes the corresponding average value.

In addition, to investigate the robustness of the extracted feature wavelengths, Pearson correlation coefficient (R), defined as Equation (5) [37], was adopted to analyze the relationships between the absorbance and adulteration content under certain feature wavelengths. Herein, Pearson correlation coefficients ranging from −1 to 0 indicate a negative correlation between adulteration content and absorbance, while the values from 0 to 1 denote a positive correlation. Moreover, the coefficients closer to 1 or −1 demonstrate a strong correlation.

(5) $$R=\frac{\sum_{i=1}^{n}{(x}_{i}-\overset{¯}{x}{)(y}_{i}-\overset{¯}{y})}{\sqrt{\sum_{i=1}^{n}{{(x}_{i}-\overset{¯}{x})}^{2}}\sqrt{\sum_{i=1}^{n}{{(y}_{i}-\overset{¯}{y})}^{2}}}$$

where x and y are the absorbance and adulteration content, respectively; $\overset{¯}{x}$ and $\overset{¯}{y}$ are the corresponding average values.

## 4. Conclusions

This study used UV-Vis-NIR transmission spectroscopy to quantitatively predict the content of soybean, rapeseed, peanut, and corn oils blended into *Camellia* oil. A transmission spectroscopic test platform was built to collect the spectra of oil samples that consisted of *Camellia* oil mixed with four different edible oils at varying ratios. First, we compared the preprocessing results produced by SG–CWT at different decomposition scales (L1–L9) and obtained SG–CWT (L5) as the best preprocessing method. After preprocessing the full spectra, we used CARS, SPA, BOSS, and IVSO algorithms to obtain the feature wavelengths. Out of these, the IVSO algorithm extracted the most helpful feature wavelengths in improving the model’s prediction accuracy. Notably, the Vis band had the largest number of these feature wavelengths. Next, four distinct quantitative models using SVR or RF models were established, respectively, for predicting the adulteration content of soybean, corn, peanut and rapeseed oils in *Camellia* oil. The results showed that SVR model was most suitable for *Camellia* oil mixed with soybean oil, while RF models were appropriate for *Camellia* oil adulterated with corn, peanut and rapeseed oils, respectively. Specifically, the SVR model for soybean oil adulteration had $R_{p}^{2}$ of 0.9925, RMSE_p_ of 0.0324 and MAE_p_ of 0.0171. Similarly, the RF models had $R_{p}^{2}$ of 0.9845, RMSE_p_ of 0.0398 and MAE_p_ of 0.0282 for corn oil adulteration, $R_{p}^{2}$ of 0.9674, RMSE_p_ of 0.0585 and MAE_p_ of 0.0267 for peanut oil adulteration, and $R_{p}^{2}$ of 0.9937, RMSE_p_ of 0.0267 and MAE_p_ of 0.0155 for rapeseed oil adulteration. Finally, the feature wavelengths proposed by IVSO were subjected to both a significance test and Pearson correlation analysis. The results showed that the correlation between adulteration content and absorbance was strongest for the Vis wavelength, followed by the NIR wavelength and then the UV wavelength. This order of correlation strength indicates that the feature wavelengths extracted by IVSO are reliable. Overall, this study provides valuable references for the development of a low-cost spectroscopic quantification device for adulterated *Camellia* oil.

## Figures and Tables

**Table 1:** Evaluation indicators of pre-processing results.

Pre-Processing Methods	Transmission Spectrum	
	$R^{2}$	RMSE
FS	0.9913	0.0310
SG	0.9913	0.0310
SG-1st	0.9973	0.0173
SG-2nd	0.9996	0.0075
SG–CWT (L1)	0.9944	0.0249
SG–CWT (L2)	0.9974	0.0173
SG–CWT (L3)	0.9989	0.0117
SG–CWT (L4)	0.9995	0.0082
SG–CWT (L5)	0.9998	0.0059
SG–CWT (L6)	0.9923	0.0290
SG–CWT (L7)	0.9902	0.0328
SG–CWT (L8)	0.9888	0.0350
SG–CWT (L9)	0.9814	0.0452

**Table 2:** The optimal parameters and evaluation criteria for SVR and RF models.

Model	Adulteration Category	Parameter Selection	Calibration Set		Prediction Set	
			$R_{c}^{2}$	${\mathrm{RMSE}}_{C}$	$R_{p}^{2}$	${\mathrm{RMSE}}_{p}$
IVSO-SVR	soybean oil	*c* =9.1896; γ=0.0039	0.9926	0.0261	0.9925	0.0325
IVSO-RF	corn oil	*n* = 17	0.9975	0.0171	0.9845	0.0398
IVSO-RF	peanut oil	*n* = 17	0.9927	0.0308	0.9674	0.0585
IVSO-RF	rapeseed oil	*n* = 14	0.9977	0.0164	0.9937	0.0267

**Table 3:** Comparison with relevant previous research results.

Authentication Categories	Spectroscopy	Range of Adulteration	$R_{P\;}^{2}$	Refs
Sesame oil adulterated with soybean oil	Vis-NIR	10–90%	0.9966	[19]
Rapeseed oil adulterated with soybean oil			0.9968	
*Camellia* oil adulterated with rapeseed oil	FTIR	0–50%	0.9805–0.9959	[22]
Extra virgin olive oil adulterated with safflower, corn, soybean, canola, sunflower and sesame oil	UV-Vis	1–20%	0.94–0.99	[15]
*Camellia* oil adulterated with soybean oil	UV-Vis-NIR	1–90%	0.9925	This study
*Camellia* oil adulterated with corn oil			0.9845	
*Camellia* oil adulterated with peanut oil			0.9674	
*Camellia* oil adulterated with rapeseed oil			0.9937	

## Data Availability

The data presented in this study are available on request from the corresponding author.

## Supplementary Materials

The following supporting information can be downloaded at: https://www.mdpi.com/article/10.3390/molecules28165943/s1, Figure S1: Research flowchart.
