# Supplementary material for: Rapid and Low-Cost Quantification of Adulteration Content in Camellia Oil Utilizing UV-Vis-NIR Spectroscopy Combined with Feature Selection Methods
Source: Molecules. 2023 Aug 8;28(16):5943. doi: 10.3390/molecules28165943 (PMC10458121; doi:10.3390/molecules28165943)
Supplement: Supplementary file 1 [file molecules-28-05943-s001.zip › molecules-2512245-supplementary.pdf]

Rapid and Low-cost Quantification of Adulteration Content in *Camellia* Oil Utilizing UV-Vis-NIR Spectroscopy Combined with Feature Selection Methods

Qiang Liu, Zhongliang Gong, Dapeng Li *, Tao Wen, Jinwei Guan and Wenfeng Zheng

School of Mechanical and Electrical Engineering, Central South University of Forestry and Technology,
Changsha, Hunan 410004, China
*Correspondence: dapengli@csuft.edu.cn

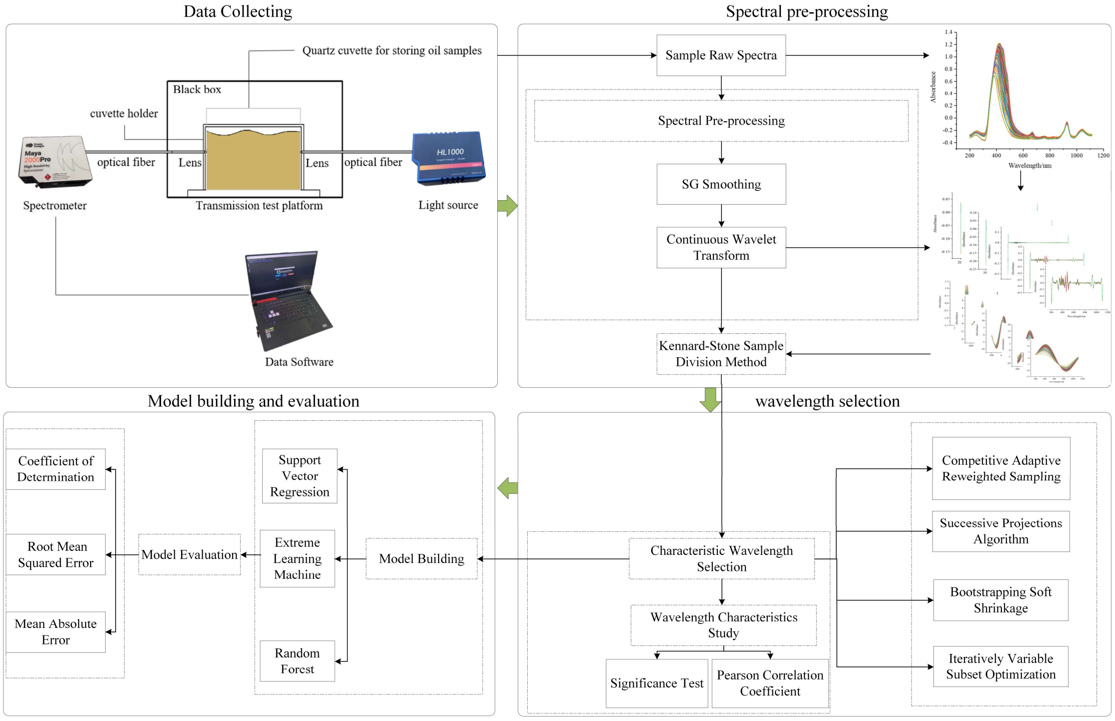

Figure S1. Research flowchart.
